# Comments on: Prenatal acetaminophen and autism: a meta-analytic signal driven by a single, mis-extracted estimate

**DOI:** 10.61622/rbgo/2026rbgo108L1

**Published:** 2026-09-23

**Authors:** Melania Maria Ramos Amorim, Alexandre Delgado, Aline Veras Morais Brilhante, Alex Sandro Rolland Souza

**Affiliations:** 1 Institute of Integral Medicine Prof. Fernando Figueira Care Center for Women Recife Pernambuco Brazil Care Center for Women, Institute of Integral Medicine Prof. Fernando Figueira, Recife, Pernambuco, Brazil.; 2 Universidade Federal de Campina Grande Campina Grande PB Brazil Universidade Federal de Campina Grande, Campina Grande, PB, Brazil.; 3 Universidade Federal do Ceará Fortaleza CE Brazil Universidade Federal do Ceará, Fortaleza, CE, Brazil.; 4 Universidade Católica de Pernambuco School of Health and Life Sciences Recife PE Brazil School of Health and Life Sciences, Universidade Católica de Pernambuco, Recife, PE, Brazil.; 5 Universidade Federal de Pernambuco Center of Medical Sciences Recife PE Brazil Center of Medical Sciences, Universidade Federal de Pernambuco, Recife, PE, Brazil.

Dear Editor,

We read with great interest the systematic review and meta-analysis by Oliveira et al.,^(1)^ which reported an 18% increase in the odds of autism spectrum disorder (ASD) following prenatal acetaminophen exposure. Because this conclusion may influence clinical practice and public health recommendations,^(2)^ several methodological aspects deserve clarification.

First, the pooled estimate for ASD diagnosis appears to be driven almost entirely by a single study contributing 95.7% of the statistical weight.^(1)^ However, the effect estimate entered for Ahlqvist et al. (OR 1.18) cannot be identified in the original publication.^(3)^ The authors reported a population-based hazard ratio (HR) of 1.05 and, more importantly, a full-sibling comparison HR of 0.98 for ASD, but no odds ratio of 1.18.^(3)^ Consequently, the estimate underlying the pooled result is not reproducible from the published data.

Second, this discrepancy changes the interpretation of the evidence. Ahlqvist et al. concluded that the modest association observed in conventional analyses was largely explained by shared familial factors, whereas the sibling-comparison analysis showed no association between prenatal acetaminophen exposure and ASD (HR 0.98).^(3)^ Therefore, the principal conclusion of the meta-analysis appears to rely on an effect estimate that neither corresponds to the published ASD results nor reflects the interpretation of the study contributing nearly all of the statistical weight.

Additional methodological issues further reduce confidence in the summary estimate. The abstract reports a 95% confidence interval that differs from the corresponding forest plot for the same pooled effect.^(1)^ Moreover, odds ratios, hazard ratios, and incidence rate ratios were combined without any reported transformation or justification. Several extracted estimates are also difficult to verify because the cited references include cohort-profile publications that do not report ASD effect estimates,^(4,5)^ while other estimates appear to have been visually extracted from figures in a previous meta-analysis^6^ or attributed to a study that investigated cryptorchidism and hypospadias rather than ASD.^(6,7)^ These approaches limit reproducibility and may introduce dependence if cohorts from a previous meta-analysis are subsequently analyzed as independent studies.

Finally, the review recommends minimizing acetaminophen prescribing during pregnancy despite the substantial risk of residual confounding by indication and shared familial factors, biases that sibling-comparison studies consistently attenuate toward the null.^(3-8)^ This recommendation is not supported by the presented evidence and is inconsistent with current guidance from major international professional societies.^(9-10)^

Given the potential implications of this review for clinical practice and public health, clarification of the extracted data and reanalysis using transparent and reproducible effect estimates would strengthen confidence in the reported conclusions.

## Data Availability

The research data are described in the letter presented.

## References

[1] Oliveira JA, Lemos MH, Queiroz LF, Sciorilli JV, Tran T, Ortiz IO (2026). Prenatal exposure to paracetamol and risk of autism spectrum disorder: systematic review and meta-analysis of observational studies. Rev Bras Ginecol Obstet.

[2] Amorim MM (2025). Paracetamol, autismo e a panaceia presidencial: quando a bravata encontra a evidência (e perde). Rev Bras Saúde Mater Infant.

[3] Ahlqvist VH, Sjöqvist H, Dalman C, Karlsson H, Stephansson O, Johansson S (2024). Acetaminophen use during pregnancy and children's risk of autism, ADHD, and intellectual disability. JAMA.

[4] Chatzi L, Leventakou V, Vafeiadi M, Koutra K, Roumeliotaki T, Chalkiadaki G (2017). Cohort profile: the mother-child cohort in Crete, Greece (Rhea Study). Int J Epidemiol.

[5] Porta D, Fantini M (2006). Prospective cohort studies of newborns in Italy to evaluate the role of environmental and genetic characteristics on common childhood disorders. Ital J Pediatr.

[6] Alemany S, Avella-García C, Liew Z, García-Esteban R, Inoue K, Cadman T (2021). Prenatal and postnatal exposure to acetaminophen in relation to autism spectrum and attention-deficit and hyperactivity symptoms in childhood: meta-analysis in six European population-based cohorts. Eur J Epidemiol.

[7] Snijder CA, Kortenkamp A, Steegers EA, Jaddoe VW, Hofman A, Hass U (2012). Intrauterine exposure to mild analgesics during pregnancy and the occurrence of cryptorchidism and hypospadia in the offspring: the Generation R Study. Hum Reprod.

[8] Gustavson K, Ystrom E, Ask H, Torvik FA, Hornig M, Susser E (2021). Acetaminophen use during pregnancy and offspring attention deficit hyperactivity disorder — a longitudinal sibling control study. JCPP Adv.

[9] American College of Obstetricians and Gynecologists (ACOG) (2025). Acetaminophen use in pregnancy and neurodevelopmental outcomes. Practice Advisory.

[10] Louwen F, Deuster E, McAuliffe FM, Jacobsson B, Geary M, Fleischman S (2025). Paracetamol (acetaminophen) use during pregnancy and autism risk: Evidence does not support causal association. Int J Gynaecol Obstet.

